# Supplementary material for: ProteinShader: illustrative rendering of macromolecules
Source: BMC Struct Biol. 2009 Mar 30;9:19. doi: 10.1186/1472-6807-9-19 (PMC2672931; doi:10.1186/1472-6807-9-19)
Supplement: Additional file 1 — ProteinShader program without source code. This compressed file contains the complete ProteinShader program including associated libraries, but no source code. A README.txt file gives an overview of the ProteinShader distribution, and the index.html file in the help subdirectory has directions on getting started with the program as well as a set of tutorials. [file 1472-6807-9-19-S1.zip › ProteinShader-beta-0_9_4-binary/help/api/org/proteinshader/graphics/Silhouette.html]

Silhouette (ProteinShader API)


|  |  |  |
| --- | --- | --- |
| **Overview** \| **Package** \| **Class** \| **Use** \| **Tree** \| **Deprecated** \| **Index** \| **Help** | |  |
| **PREV CLASS**   **NEXT CLASS** | **FRAMES**    **NO FRAMES**     **All Classes** |  |
| SUMMARY: NESTED \| FIELD \| CONSTR \| METHOD | DETAIL: FIELD \| CONSTR \| METHOD |  |


---


## org.proteinshader.graphics Class Silhouette

```
java.lang.Object
  org.proteinshader.graphics.Silhouette
```

---

``` public class Silhouette extends Object ```

Uses jitter to repeatedly render a translucent black silhouette of a Drawable
object for the purpose of antialiasing (smoothing jagged edges).

---

| **Field Summary** | |
| --- | --- |
| `static boolean` | `DEFAULT`             Determines if translucent black silhouettes are used by default for antialiasing the dark edges of tubes or ribbons drawn with halftoning. |
| `static double[]` | `JITTER_4`             Holds (xPixels, yPixels) values for translating 4 times with jitter. |


**Constructor Summary**

`Silhouette(ShaderManager shaders)`
  
          Constructs a Silhouette object.


| **Method Summary** | |
| --- | --- |
| `void` | `drawRibbon(GL gl, Segment s, SegmentListInfo info, boolean extraLines)`             Draws a translucent black silhoutte of a ribbon segment using jitter. |
| `void` | `drawTube(GL gl, Segment s, SegmentListInfo info, boolean extraLines)`             Draws a translucent black silhoutte of a tube segment using jitter. |

**Methods inherited from class java.lang.Object**

`clone, equals, finalize, getClass, hashCode, notify, notifyAll, toString, wait, wait, wait`

**Field Detail**

### DEFAULT

```
public static final boolean DEFAULT
```

:   Determines if translucent black silhouettes are used by default
    for antialiasing the dark edges of tubes or ribbons drawn with
    halftoning.

    **See Also:**: Constant Field Values

---


### JITTER\_4

```
public static final double[] JITTER_4
```

:   Holds (xPixels, yPixels) values for translating 4 times with jitter.


**Constructor Detail**

### Silhouette

```
public Silhouette(ShaderManager shaders)
```

:   Constructs a Silhouette object.


**Method Detail**

### drawTube

```
public void drawTube(GL gl,
                     Segment s,
                     SegmentListInfo info,
                     boolean extraLines)
```

:   Draws a translucent black silhoutte of a tube segment using jitter.

    :   **Parameters:**: `gl` - the current GL object.: `s` - the Segment to draw.: `info` - holds names of OpenGL display lists for the Segment.: `extraLines` - indicates if extra lines are drawn at start and end.

---


### drawRibbon

```
public void drawRibbon(GL gl,
                       Segment s,
                       SegmentListInfo info,
                       boolean extraLines)
```

:   Draws a translucent black silhoutte of a ribbon segment using jitter.

    :   **Parameters:**: `gl` - the current GL object.: `s` - the Segment to draw.: `info` - holds names of OpenGL display lists for the Segment.: `extraLines` - indicates if extra lines are drawn at start and end.


---


|  |  |  |
| --- | --- | --- |
| **Overview** \| **Package** \| **Class** \| **Use** \| **Tree** \| **Deprecated** \| **Index** \| **Help** | |  |
| **PREV CLASS**   **NEXT CLASS** | **FRAMES**    **NO FRAMES**     **All Classes** |  |
| SUMMARY: NESTED \| FIELD \| CONSTR \| METHOD | DETAIL: FIELD \| CONSTR \| METHOD |  |


---

# *Copyright © 2007-2008*
